# Complete genome sequence of *Lactiplantibacillus plantarum* strain VHProbi P06, isolated from kimchi

**DOI:** 10.1128/MRA.00552-23

**Published:** 2023-10-31

**Authors:** Chaoqun Guo, Hongchang Cui, Qian Wang, Zhi Duan

**Affiliations:** 1 Qingdao Vland biotech Group Co., Ltd, Qingdao, China; University of Maryland School of Medicine, Baltimore, Maryland, USA

**Keywords:** complete genome sequence, *Lactiplantibacillus plantarum*, VHProbi P06

## Abstract

*Lactiplantibacillus plantarum* strain VHProbi P06 is a probiotic that was isolated from kimchi soup. Here, we investigate the whole-genome sequence of this strain, which contains a chromosome and seven plasmids.

## ANNOUNCEMENT

One milliliter of kimchi soup purchased from the supermarket was diluted in 99 mL of de Man-Rogosa-Sharpe (MRS) agar (Haibo, China) broth medium. Then, 100 µL of dilution was spread onto the MRS agar plate and incubated under anaerobic conditions at 37°C for 24 h to enrich bacteria. A colony named VHProbi P06 was picked up and identified as the species *Lactiplantibacillus plantarum* using 16S rRNA sequencing, as described by Liu et al. (1). The Illumina HiSeq 2500 and the PacBio RS II Single Molecule Real Time (SMRT) sequencing platforms were combined to sequence the genome of this strain. The strain was cultured in the same growth condition as isolation for DNA extraction. Total DNA was extracted using the Genomic DNA Purification Kit (Promega, USA) following its instruction and was used for the preparation of both the Illumina and the PacBio libraries. Template DNA was sheared into 400-bp fragments for the Illumina library creation using the TruSeq library preparation kit from Illumina. The sheared fragments were used to prepare the Illumina libraries using the NEXTflex Rapid DNA-Seq Kit (BioScientific, USA). The prepared libraries were then used for paired-end Illumina sequencing (2 × 150 bp). For the PacBio sequencing, DNA was spun in a Covaris G-Tube (Covaris, Woburn, MA, USA) to be sheared into fragments. Then, ~10-kb fragments were purified, end-repaired, and ligated with the SMRTbell sequencing adapters following the PacBio recommendation. Next, a ~10-kb insert library was prepared using SMRTbell Express Template Prep Kit v.2.0 and sequenced on one SMRT cell using standard methods. Finally, 8,091,694 raw reads and 7,991,932 clean reads were generated using the Illumina sequencing platform to reach a depth of 1165.49-fold coverage and 385,591 raw reads (*N*_50_; 218,077 bp) were generated using the PacBio sequencing platform. For Illumina raw data, adapters, non-A, G, C, and T bases of the 5′ end reads containing >10% unknown *N* and low-quality reads were removed by using Sickle v.1.33 (https://github.com/najoshi/sickle). Clean data were assembled into a scaffold using SOAPdenovo v.2.04 (2). The PacBio raw reads were then assembled into a scaffold using Hierarchical Genome Assembly Process v.4.0 (3) and Canu v.2.2 (4). Error correction of the PacBio assembly results was performed by Pilon v.1.22 (5) using the Illumina reads. If there was an overlap at the two ends with 5,000 bp, one end of the overlap was cut off to circulate the sequences. The final assembly generated a complete genome with a seamless chromosome and seven plasmids. Glimmer v.3.02 (6), tRNA-scan-SE v.2.0 (7), and Barrnap v.0.9 were used to predict protein-coding genes, tRNA and rRNA, respectively (https://github.com/tseemann/barrnap/). CheckM v.1.1.3 was used to check the quality of the assembled genome sequence, with a completeness of 99.45%, contamination of 0%, and strain heterogeneity of 0% (8). Genome annotations were conducted using the National Center for Biotechnology Information Prokaryotic Genome Annotation Pipeline v.5.3 (9). Default parameters were used for all software unless otherwise specified.

The whole-genome sequence of *L. plantarum* VHProbi P06 contains a 3,206,682-bp circular chromosome with GC contents of 44.66%. There were 3,115 protein-coding genes, 16 rRNA genes (5S rRNA, 16S rRNA, and 23S rRNA), 69 tRNA genes, 4 ncRNA genes, and 63 pseudogenes in the genome. There are also seven plasmids (Table 1). We stated that these molecules are plasmids verified by Pilon v.1.22.

**TABLE 1:** Summary data of the plasmids

Plasmid name	Size (bp)	G+C (%)	GenBank accession no.
pPAP06	63,957	40.48	CP104085
pPBP06	35,676	39.53	CP104086
pPCP06	33,304	39.43	CP104087
pPDP06	29,305	36.69	CP104088
pPEP06	25,244	40.01	CP104089
pPFP06	14,030	38.39	CP104090
pPGP06	2,360	35.97	CP104091

## Data Availability

The 16S rRNA sequence and the whole-genome sequence of *Lactiplantibacillus plantarum* VHProbi P06 have been deposited in the database with GenBank accession numbers OQ592686 and CP104084 to CP104091, SRA accession numbers SRR21288989 and SRR21288990, BioProject accession number PRJNA874163, and BioSample accession number SAMN30526907.
